# Engagement With Daily Symptom Reporting, Passive Smartphone Sensing, and Wearable Device Data Collection During Chemotherapy: Longitudinal Observational Study

**DOI:** 10.2196/57347

**Published:** 2024-12-10

**Authors:** Sean McClaine, Jennifer Fedor, Christianna Bartel, Leeann Chen, Krina C Durica, Carissa A Low

**Affiliations:** 1 University of Pittsburgh Pittsburgh, PA United States

**Keywords:** cancer, chemotherapy, remote monitoring, mobile health, wearable device, mobile phone, oncology, metastases, chemo, mHealth, mobile application, digital health, digital intervention

## Abstract

**Background:**

Chemotherapy can cause symptoms that impair quality of life and functioning. Remote monitoring of daily symptoms and activity during outpatient treatment may enable earlier detection and management of emerging toxicities but requires patients, including older and acutely ill patients, to engage with technology to report symptoms through smartphones and to charge and wear mobile devices.

**Objective:**

This study aimed to identify factors associated with participant engagement with collecting 3 data streams (ie, daily patient-reported symptom surveys, passive smartphone sensing, and a wearable Fitbit device [Google]) during chemotherapy.

**Methods:**

We enrolled 162 patients receiving outpatient chemotherapy into a 90-day prospective study. Patients were asked to install apps on their smartphones to rate daily symptoms and to collect passive sensor data and to wear a Fitbit device for the duration of the study. Participants completed baseline demographic and quality of life questionnaires, and clinical information was extracted from the electronic medical record. We fit a series of logistic generalized estimating equations to evaluate the association between demographic and clinical factors and daily engagement with each data stream.

**Results:**

Participants completed daily surveys on 61% (SD 27%) of days and collected sufficient smartphone data and wearable sensor data on 73% (SD 35%) and 70% (SD 33%) of enrolled days, respectively, on average. Relative to White participants, non-White patients demonstrated lower odds of engagement with both symptom surveys (odds ratio [OR] 0.49, 95% CI 0.29-0.81; *P*=.006) and wearable data collection (OR 0.35, 95% CI 0.17-0.73; *P*=.005). Patients with stage 4 cancer also exhibited lower odds of engagement with symptom reporting than those with earlier stage disease (OR 0.69, 95% CI 0.48-1.00; *P*=.048), and patients were less likely to complete symptom ratings on the weekend (OR 0.90, 95% CI 0.83-0.97; *P*=.008). Older patients (OR 1.03, 95% CI 1.01-1.06; *P*=.01) and those who reported better cognitive functioning at study entry (OR 1.18, 95% CI 1.03-1.34; *P*=.02) were more likely to engage with Fitbit data collection, and patients who reported higher levels of depressive symptoms were less likely to engage with smartphone data collection (OR 1.18, 95% CI 1.03-1.36; *P*=.02).

**Conclusions:**

Remote patient monitoring during chemotherapy has the potential to improve clinical management, but only if patients engage with these systems. Our results suggest significant associations between demographic and clinical factors and long-term engagement with smartphone and wearable device assessments during chemotherapy. Non-White participants, those with metastatic cancer, or those with existing cognitive impairment may benefit from additional resources to optimize engagement. Contrary to hypotheses, older adults were more likely than younger adults to engage consistently with wearable device assessments.

## Introduction

Patients with cancer undergoing chemotherapy often experience numerous adverse effects, including fatigue, nausea and vomiting, peripheral neuropathies, and more [1]. These symptoms can have a significant negative impact on the patient’s quality of life and can lead to early discontinuation or reduction of treatment.

Growing evidence suggests that patients who used symptom-reporting software during chemotherapy continued their treatment for longer, required fewer hospital admissions, and survived longer than those who were not randomized to report symptoms [2-4]. Symptom monitoring systems associated with improved clinical outcomes use patient-generated data to trigger alerts to clinicians and to enable the treating oncology team to manage symptoms earlier. To achieve these potential benefits, patients, including those who are older, acutely ill, or with low digital or health literacy, must engage with technological systems to report symptoms and provide other patient-generated health data for remote monitoring purposes. This paper’s goal is to characterize patient engagement with a system aimed at capturing daily patient-reported symptoms and continuous wearable and smartphone sensor data during chemotherapy.

Smartphones and other technologies provide a unique opportunity for remote patient monitoring as they allow patients to record their symptoms and other patient-reported outcomes quickly and easily. Clinicians can benefit from patients electronically recording and sharing their symptoms, as they can use this information to track their patient’s symptom progression and identify concerning symptoms in real time. Several studies have investigated patient adherence to daily or weekly symptom surveys on the patient’s smartphone or by email [5-9]. Typical adherence rates in the literature have varied depending on the technology used, the frequency and duration of assessments, how adherence is defined, and whether participants were given reminders to answer symptom surveys. A systematic review of 33 different electronic symptom self-reporting systems reported response rates ranging from 45% to 92% [5].

Wearable devices such as Fitbits (Google) and other activity monitors as well as passive data from smartphones may also be useful for patient monitoring, as they allow for the continuous collection of physiological and behavioral data related to sleep, activity, geographic mobility, and more. These data may also be helpful to clinicians, as studies have shown a correlation between lower step counts and negative patient outcomes including greater symptom burden, lower quality of life and performance status, and worse clinical outcomes among oncology patients [10-12]. The growing literature in this area suggests that patient adherence to wearable data collection during cancer treatment has been relatively robust [13,14]. A systematic review of 38 studies that investigated adherence of patients with cancer to wearable devices reported adherence rates ranging from 60%-100% [14]. Collecting data from a wearable device may require less active involvement from participants but requires the participant to keep the device charged, wear it consistently and correctly, and sync the wearable to an internet-connected device. Indeed, there is evidence that patient adherence to wearable devices may be limited when the patient is not given reminders to wear and sync the device [15]. Other barriers to wearable device data collection reported in the literature include limited technical literacy and limited access to a reliable internet connection [16]. Passive smartphone sensor data collection is less common, and to our knowledge, no studies to date have examined patient adherence to passive smartphone sensing during chemotherapy. In addition, there has been little research done on the sociodemographic and medical factors that affect a participant’s engagement with these technology-based monitoring systems during cancer treatment.

The objective of this study was to identify factors that impacted participant engagement with collecting 3 data streams over 90 days during chemotherapy, that is, daily patient-reported symptom surveys, passive smartphone sensing, and a wearable Fitbit device.

## Methods

### Participants

Potential study participants were identified for the study by their medical oncology care team. Men and women aged 18 years or older who were undergoing chemotherapy for any solid tumor at a large academic cancer center, who owned a smartphone, who could read and write in English, and who had at least 2 chemotherapy cycles remaining were eligible to participate. In addition, 7 participants were recruited from a community research registry, and these participants were asked to self-report on if they met the above eligibility criteria.

### Ethical Considerations

The institutional review board of the University of Pittsburgh reviewed and approved all study activities (study 19070011). The study team conducted informed consent by explaining each study app, what specific data passive sensors would collect, how the data from the Fitbit and mobile apps would be used for the purpose of the study, how information was deidentified, potential risks, and asking participants for permission to install each study app on their phone. All data were stored in secure locations and identified only by anonymized study ID numbers. Participants were compensated US $100 and given the option to keep the Fitbit (approximate value US $100) upon completion of the study.

### Study Procedure

First, participants had the MoSHI Surveys app (Carissa Low) installed on their smartphones; this free commercially available app was developed by our research team and is used to configure notifications to remind study participants to complete web-based surveys. This app delivered a daily and weekly (weekly data not reported) symptom survey. We focused on daily symptom surveys given that daily symptom assessments are more burdensome to participants but also potentially beneficial for capturing rapidly developing symptoms sooner [17]. The daily survey asked about symptoms experienced in the past 24 hours, was based on the National Cancer Institute’s Patient Reported Outcome-Common Terminology Criteria for Adverse Events [18], and included the following symptoms, selected to represent common side effects of cytotoxic chemotherapy: nausea, vomiting, decreased appetite, abdominal pain, constipation, diarrhea, shortness of breath, insomnia, fatigue, rash, dizziness, numbness or tingling in hands or feet, anxiety, sad or unhappy feelings, and “other symptoms.” Participants were able to set times for daily notifications to remind them to complete the surveys. These notifications would occur once a day at the set time, and alert sounds and other settings were determined by the participant’s notification settings for their phone.

The AWARE app (developed by Denzil Ferreira and Yuuki Nishiyama) [19], another free and commercially available app developed by our research collaborators, was also installed on participants’ Android (Google) or iOS (Apple Inc) smartphones. AWARE runs in the background to record information about movement and location of the phone, screen on and off events, nearby Bluetooth devices and Wi-Fi networks, and metadata about calls and SMS text messages exchanged using the smartphone. Participants were asked to keep the app open and running in the background of their phones for the duration of the study. Finally, participants were provided with a Fitbit Inspire device that recorded the patient’s activity, heart rate, and sleep patterns, and the Fitbit app was installed on their smartphone to enable frequent syncing with the wearable device and upload of data to our research server. Participants were asked to wear the Fitbit at all times except when charging (approximately every 10 days). After installation and setup, a study team member taught each participant how to use all study apps and Fitbit (ie, how to change notification settings, sync their Fitbit device with their phone, view data, and so on).

Data were collected from each participant for 3 months. Incoming data quality was monitored with a secure web-based study dashboard throughout the study. The study dashboard had a column for each of the data sources, and a flag would appear after 3 consecutive days without data from a participant. This dashboard was reviewed at least 3 times a week by study team members. Generally, the participant would be contacted through phone, text, email, or in person according to their preferred method of communication and treatment schedule. If the flag remained for over a week, the participant would be called or visited in person at their next treatment. If the participant did not respond after 3 contacts, we would continue to attempt to reach out every 1 to 2 weeks if the flag remained. All communication with the participants was logged in a record of communication containing pertinent notes that all study team members had access to and updated. There was some subjective judgment around when or if a participant was contacted based on notes from previous contacts (eg, if participants were very sick, if they were receiving surgery, and if they were hospitalized).

At baseline, participants completed a demographic questionnaire as well as the Patient-Reported Outcomes Measurement Information System Profile (PROMIS-29+2 v2.1). Information about participants’ cancer and its treatment was extracted from the electronic medical record (EMR).

### Measures

#### Demographics

Demographic variables were self-reported by participants in a baseline questionnaire and included age (in years), gender or sex (male, female, and non-binary), race (White or Caucasian, Black or African American, Asian, other, more than 1 race), highest level of education (less than a high-school diploma, high-school diploma or equivalent, some college but no degree, Associates of arts or other 2-year degree, Bachelor’s degree, and Graduate degree). Residential zip code was used to classify participants as rural (yes or no) based on eligible zip code data from the Federal Office of Rural Health Policy [20]. Smartphone model information was recorded by the study team and verified against data collected by AWARE. Phone type was categorized as iOS if the device brand was “iPhone” (Apple Inc) and as Android otherwise.

#### Clinical

Insurance plan type was extracted from the EMR in June 2023 and categorized by the study team as public; private; mixed public, private, or other; or none, if no insurance was listed. Because we were unable to determine if a lack of available insurance information was due to the participant not having insurance coverage, removal of insurance information from the system upon death, or another reason, we subsequently chose to treat no insurance listed as missing. Cancer type (biliary, bone, breast, gastrointestinal tract, gynecologic, liver, lung, multiple myeloma, pancreas, salivary gland, and urogenital), stage (0, 1, 2, 3, and 4), and diagnosis date were extracted from the EMR at enrollment. For consistency, the cancer diagnosis date was defined as the date listed beside the cancer type in the participant’s outpatient progress notes. Time in days since cancer diagnosis at enrollment was calculated by subtracting the cancer diagnosis date from the study enrollment date and was rescaled to time in months for interpretability of analyses.

#### Quality of Life

To assess quality of life, participants completed the PROMIS Profile 29+2 v2.1 [21] as part of the baseline questionnaire. From each participant’s item-level responses, we obtained domain-level theta values from the HealthMeasures Scoring Service [22] and used these values to generate PROMIS preference-based scores [23]. Theta values from the pain interference, cognitive function, depression or sadness, ability to participate in social roles or activities, anxiety or fear, fatigue, physical function, and sleep disturbance domains were used to compute one overall (“PROPr [PROMIS-Preference scoring system]”) and 7 domain-specific preference-based scores. Possible scores range from 0 (reflecting death) to 1 (reflecting full health).

#### Time-Related

Time-varying, day-level variables included an index for study day (with 0 corresponding to the date of enrollment), an indicator for weekday or weekend days, and the time in days since the participant’s last known chemotherapy treatment. Dates on which the participant received chemotherapy treatment were extracted from the EMR where available. For each day for each participant, we computed the number of days that had elapsed since the participant’s last known chemotherapy treatment as the difference in days between the study day date and the most recent previous treatment date; the value of this variable was 0 on treatment days and was missing on days before the participant’s first known treatment day.

#### Daily Symptom Survey Completion

To evaluate associations between demographic, clinical, quality of life, and time-related factors, and adherence to daily surveys, we created a day-level, binary outcome variable reflecting daily symptom survey completion. For each day for each participant, adherence to daily symptom survey completion was defined as the presence of a recorded survey response that was started at any time on the given day and was at least 50% complete. This threshold was selected based on the literature [24,25].

#### Smartphone and Fitbit Data Collection

To evaluate associations between demographic, clinical, quality of life, and time-related factors and adherence to smartphone and Fitbit data collection, we created separate day-level, binary outcome variables reflecting the presence of at least 8 valid hours of phone or Fitbit data, respectively. This threshold was also based on the literature as well as our previous work [26-28]. We first used our Reproducible Analysis Pipeline for Data Streams (RAPIDS) [29] to extract day-level (24 hours from midnight to midnight) phone and Fitbit data yield features for each participant. Data yield features approximate the proportion of each day during which the device was sensing data from any of the specified sensors. For each day for each participant, adherence to phone data collection was defined as at least 8 valid hours of data from any AWARE sensor (activity recognition, app crashes, apps foreground, apps notifications, battery, Bluetooth, calls, keyboard, light, locations, SMS text messages, screen, Wi-Fi–connected, and Wi-Fi–visible), and adherence to Fitbit data collection was defined as at least 8 valid hours of Fitbit intraday heart rate data. Valid hours were defined as 60-minute windows in which at least 1 row of raw data from any of the specified sensors was recorded in at least 30 of those minutes.

### Statistical Analysis

We first computed descriptive statistics of demographic, clinical, quality of life, and time-related measures to characterize our sample. For continuous variables, Wilcoxon rank sum tests, and for categorical variables, chi-square or Fisher exact tests were used to determine if these measures significantly differed between participants who completed the full study protocol and those who withdrew early. In addition, to characterize overall adherence in our sample, for each participant, we calculated the proportions of days with adherence to daily symptom survey completion, smartphone data collection, and Fitbit data collection as the ratio between the respective number of adherent days and the number of days the participant was enrolled in the study and computed descriptive statistics. For statistical models, we evaluated the day-level, binary outcomes.

For interpretability of analyses, age was centered at the mean age of the sample. Due to low frequencies of some categories, nonbinary gender was treated as missing, and race and highest level of education were collapsed into binary variables (respectively, White or Caucasian, not White or Caucasian; less than a college degree, college degree or higher). In addition, cancer types with frequency <10 were collapsed into a single other category, and the cancer stage was collapsed into a binary variable representing stage 4 cancer (yes or no). Baseline PROMIS preference-based scores were rescaled for interpretability by multiplying each score by 10.

To evaluate the associations between demographic, quality of life, clinical, and time-related factors and daily adherence to daily survey completion and smartphone and Fitbit data collection, we first fit a series of univariable logistic generalized estimating equations (GEE) [30] using the geepack package for R (v1.3.9; R Core Team) [31], with each binary, day-level outcome as the dependent variable and, separately, each factor as the independent variable. Due to a small proportion of missing values for some predictors, we analyzed model-wise complete cases. Because phone data yield was systematically lower among participants using Android devices compared with those using iOS devices due to differences in sensor data sampling frequencies across platforms, all models for the phone data yield outcome were additionally adjusted for phone type. GEE is a method for modeling clustered data, such as those from a longitudinal study, where observations within a cluster (ie, participants) are correlated. Either an exchangeable or first-order autoregressive (ar1) working correlation structure was selected by minimizing the quasi-information criterion (QIC). Robust SEs for parameter estimates were obtained using the sandwich estimator. Estimates were exponentiated to obtain odds ratios (OR) and 95% CIs. Because likelihood-based methods are not available for GEE, we used a series of Wald tests to conduct single- and multi-parameter inference. We accounted for multiple comparisons for each outcome by controlling for the false discovery rate [32] when evaluating global predictor effects across univariable models (*Q* values). An α level of .05 was used as a strict cutoff for determining statistical significance.

Finally, for each outcome, we fit a single multivariable GEE containing a purposefully selected subset of predictors which were determined a priori. For the sufficient Fitbit data yield outcome, we defaulted to an independent working correlation structure because unstable and extreme parameter estimates were obtained under both exchangeable and ar1 correlation structures; an ar1 correlation structure was selected for all other outcomes based on QIC, with the exception of an exchangeable working correlation structure for the sufficient phone data yield outcome.

All analyses were performed using R (v4.2.3) [33]. All code for data management and analysis is available on GitHub [34].

## Results

### Participant Characteristics

Of the 320 potential participants approached about the study through March 8, 2023, a total of 167 (52.2%) participants enrolled. Reasons for not participating in the study included concerns about technology, feeling overwhelmed, being too busy, not feeling well, and not being interested. Data collection for this prospective cohort study is ongoing; this analysis focuses on 162 patients who had completed (146/162, 90.1%) or withdrawn from (16/162, 9.9%) the 90-day study protocol between March 2020 and June 2023. Participant characteristics are summarized in Table 1. Participants were aged 59.47 (SD 11.84, range 28-92) years on average, and were mostly female (101/162, 62.3%), White or Caucasian (135/162, 83.3%), had obtained a bachelor’s degree (42/162, 25.9%), did not live in a rural zip code (145/162, 89.5%), and used an iOS smartphone (98/162, 60.5%). Most participants had a private insurance plan (79/162, 48.8%), gastrointestinal tract cancer (57/162, 35.2%), stage 4 cancer (103/162, 63.6%), and enrolled in the study 10.88 (SD 22.01, range 0-124) months after their cancer diagnosis, on average. Furthermore, 1 participant enrolled through the community research registry was diagnosed with multiple myeloma rather than a solid tumor. With the exception of insurance plan type (*P*=.02), participant characteristics did not significantly differ between participants who completed the study and those who withdrew early (all *P*>.08). Participants were enrolled in the study for a grand total of 13,954 days, with an average of 86 (SD 17, range 8-92) days per participant. Day-level characteristics are summarized in Table 2.

**Table 1:** Participant characteristics.

Characteristic		Study completion status				
		Overall, N=162	Completed, n=146	Withdrawn, n=16	*P* value^a^	
Age (years), mean (SD)		59.47 (11.84)	59.97 (11.90)	54.94 (10.54)	.11	
**Sex, n (%)**						.09
	Female	101 (62.3)	87 (59.6)	14 (88)		
	Male	60 (37.0)	58 (39.7)	2 (13)		
	Nonbinary	1 (0.6)	1 (0.7)	0 (0)		
**Race, n (%)**						.71
	White or Caucasian	135 (83.3)	122 (83.6)	13 (81)		
	Black or African American	21 (13)	18 (12.3)	3 (19)		
	Asian	1 (0.6)	1 (0.7)	0 (0)		
	Other	2 (1.2)	2 (1.4)	0 (0)		
	More than 1 race	3 (1.9)	3 (2.1)	0 (0%)		
**Ethnicity, n (%)**						.34
	Non-Hispanic	158 (97.5)	143 (97.9)	15 (94)		
	Hispanic	1 (0.6)	1 (0.7)	0 (0.0)		
	Unknown	3 (1.9)	2 (1.4)	1 (6)		
**Education, n (%)**						.09
	Less than a high-school diploma	2 (1.2)	1 (0.7)	1 (6)		
	High-school diploma or equivalent	32 (19.8)	30 (20.5)	2 (13)		
	Some college but no degree	32 (19.8)	28 (19.2)	4 (25)		
	Associate of arts or other 2-year degree	15 (9.3)	13 (8.9)	2 (13)		
	Bachelor’s degree	42 (25.9)	37 (25.3)	5 (31)		
	Graduate degree	37 (22.8)	36 (24.7)	1 (6)		
	Unknown	2 (1.2)	1 (0.7)	1 (6)		
**Rural zip code, n (%)**						.38
	No	145 (89.5)	132 (90.4)	13 (81)		
	Yes	17 (10.5)	14 (9.6)	3 (19)		
**Phone type, n (%)**						.71
	iPhone	98 (60.5)	89 (61)	9 (56)		
	Android	64 (39.5)	57 (39)	7 (44)		
**Baseline PROMIS^b^ preference score, mean (SD)^c^**						
	PROPr^d^	0.43 (0.23)	0.43 (0.23)	0.38 (0.19)	.51	
	Cognition	0.83 (0.20)	0.83 (0.20)	0.85 (0.13)	>.99	
	Depression	0.88 (0.15)	0.89 (0.13)	0.80 (0.28)	.26	
	Fatigue	0.77 (0.15)	0.76 (0.15)	0.79 (0.12)	.57	
	Pain	0.85 (0.21)	0.85 (0.21)	0.82 (0.16)	.23	
	Physical	0.76 (0.18)	0.76 (0.18)	0.77 (0.18)	.63	
	Sleep	0.77 (0.16)	0.77 (0.16)	0.76 (0.13)	.56	
	Social	0.79 (0.18)	0.79 (0.18)	0.78 (0.17)	.55	
**Insurance plan type, n (%)**						.02
	Private	79 (48.8)	68 (46.6)	11 (69)		
	Public	51 (31.5)	49 (33.6)	2 (13)		
	Mixed	21 (13)	21 (14.4)	0 (0)		
	Unknown	11 (6.8)	8 (5.5)	3 (19)		
**Cancer type, n (%)**						.67
	Biliary	7 (4.3)	7 (4.8)	0 (0)		
	Bone	1 (0.6)	1 (0.7)	0 (0)		
	Breast	24 (14.8)	23 (15.8)	1 (6)		
	Gastrointestinal tract	57 (35.2)	49 (33.6)	8 (50)		
	Gynecologic	9 (5.6)	7 (4.8)	2 (13)		
	Liver	2 (1.2)	2 (1.4)	0 (0)		
	Lung	6 (3.7)	6 (4.1)	0 (0)		
	Multiple myeloma	1 (0.6)	1 (0.7)	0 (0)		
	Pancreas	40 (24.7)	35 (24)	5 (31)		
	Salivary gland	1 (0.6)	1 (0.7)	0 (0)		
	Urogenital	14 (8.6)	14 (9.6)	0 (0)		
**Cancer stage, n (%)**						.81
	0	1 (0.6)	1 (0.7)	0 (0)		
	1	10 (6.2)	10 (6.8)	0 (0)		
	2	25 (15.4)	23 (15.8)	2 (13)		
	3	20 (12.3)	19 (13)	1 (6)		
	4	103 (63.6)	90 (61.6)	13 (81)		
	Unknown	3 (1.9)	3 (2.1)	0 (0)		
Time since diagnosis (months), mean (SD)		10.88 (22.01)	11.69 (23.00)	3.50 (4.62)	.15	

^a^Wilcoxon rank sum test; Fisher exact test; Pearson chi-square test.

^b^PROMIS: Patient-Reported Outcomes Measurement Information System.

^c^Data missing for 3/162 participants (1.8%).

^d^PROPr: PROMIS-Preference scoring system.

**Table 2:** Day-level characteristics.

Characteristic		N=13,954
Study day, mean (SD), (range)		44.18 (26.28), (0-91)
**Weekend, n (%)**		
	No	9976 (71.49)
	Yes	3978 (28.51)
Time since last chemotherapy (days), mean (SD), (range)^a^		11.21 (12.04), (0-90)

^a^Data missing for 1257/13954 days (9.01%).

### Overall Adherence

Across participants, 41.7% (5816/13,954) of days had valid data from all 3 data streams; 33.6% (4694/13,954) had valid data from 2 data streams (1090/4694, 23.2% daily survey and smartphone, 1417/4694, 30.2% daily survey and Fitbit, and 2187/4694, 46.6% smartphone and Fitbit), 17.1% (2391/13,954) had valid data from a single data stream (449/2391, 18.8% daily survey only, 1257/2391, 52.6% smartphone only, and 685/2391, 28.6% Fitbit only), and 7.6% (1053/13,954) had valid data from no data streams. Overall adherence was higher for passive smartphone and Fitbit data streams than for patient-reported daily symptom surveys (Table 3). On average, participants were adherent to daily survey completion on 60.96% (SD 27.24%, range 0%-100%), smartphone data collection on 73.06% (SD 34.94%, range 0%-100%), and Fitbit data collection on 70.07% of enrolled days (SD 33.45%, range 0%-100%).

**Table 3:** Descriptive statistics of overall adherence.

Outcome	N=162, mean (SD), (range)^a^
Daily survey adherence	60.96 (27.24), (0-100)
Smartphone adherence	73.06 (34.94), (0-100)
Fitbit adherence	70.07 (33.45), (0-100)

^a^Percent of enrolled days per participant.

On average, participants included in analyses were contacted 3.67 times throughout the duration of the study with a range of 0-12 contacts per participant and the majority of contacts taking place over text. No participants had to be withdrawn due to complete noncompliance.

### Univariable Models

Results of the univariable models characterizing associations between each demographic, quality of life, clinical, and time-related factor and daily adherence to daily survey completion and smartphone and Fitbit data collection are summarized in Table 4.

**Table 4:** Summary of results of univariable generalized estimating equations.

Predictor^a^		N	Daily survey adherence				Smartphone adherence				Fitbit adherence		
			OR (95% CI)^b^	*P* value^c^	*Q* value^d^	OR (95% CI)^b^		*P* value^c^	*Q* value^d^	OR (95% CI)^b^		*P* value^c^	*Q* value^d^
Age (years, centered at mean)		13,954	1.01 (0.99-1.02)	.34	.51	1.00 (0.97-1.02)		.81	.94	1.02 (1.00-1.05)		.03	.13
**Sex**		13,863	—^e^	.55	.67	—		.32	.51	—		.92	.95
	Female	8485	Reference	—	—	Reference		—	—	Reference		—	—
	Male	5378	0.89 (0.62-1.29)	.55	—	1.35 (0.75-2.42)		.32	—	1.03 (0.62-1.71)		.92	—
**Race (collapsed)**		13,954	—	.004	.02	—		.91	.94	—		.002	.02
	White or Caucasian	11,631	Reference	—	—	Reference		—	—	Reference		—	—
	Not White or Caucasian	2323	0.48 (0.29-0.80)	.004	—	1.04 (0.54-2.00)		.91	—	0.36 (0.19-0.68)		.002	—
**Education (collapsed)**		13,820	—	.41	.54	—		.57	.75	—		.15	.39
	College degree or higher	6883	Reference	—	—	Reference		—	—	Reference		—	—
	Less than college degree	6937	0.86 (0.61-1.22)	.41	—	0.85 (0.49-1.48)		.57	—	0.70 (0.43-1.14)		.15	—
**Rural zip code**		13,954	—	.80	.88	—		.94	.94	—		.88	.95
	No	12,510	Reference	—	—	Reference		—	—	Reference		—	—
	Yes	1444	1.08 (0.61-1.92)	.80	—	1.02 (0.60-1.73)		.94	—	0.94 (0.45-2.00)		.88	—
**Phone type**		13,954	—	.85	.89	—		<.001	<.001	—		.10	.30
	iPhone	8433	Reference	—	—	Reference		—	—	Reference		—	—
	Android	5521	0.96 (0.66-1.40)	.85	—	0.07 (0.04-0.12)		<.001	—	0.65 (0.40-1.08)		.10	—
Baseline PROMIS^f^, PROPr^g^		13,756	1.08 (1.00-1.17)	.06	.25	1.07 (0.95-1.21)		.28	.49	1.06 (0.94-1.19)		.35	.58
Baseline PROMIS, cognition		13,756	0.98 (0.89-1.06)	.57	.67	0.92 (0.81-1.04)		.18	.49	1.14 (1.00-1.31)		.05	.19
Baseline PROMIS, depression		13,756	1.06 (0.96-1.16)	.25	.41	1.14 (1.00-1.29)		.046	.32	1.11 (0.94-1.31)		.21	.44
Baseline PROMIS, fatigue		13,756	1.07 (0.96-1.19)	.24	.41	1.11 (0.95-1.30)		.19	.49	1.08 (0.91-1.28)		.40	.59
Baseline PROMIS, pain		13,756	1.07 (0.99-1.17)	.10	.34	1.11 (0.98-1.25)		.09	.49	1.06 (0.95-1.19)		.31	.58
Baseline PROMIS, physical		13,756	1.06 (0.96-1.16)	.25	.41	1.09 (0.94-1.26)		.27	.49	1.00 (0.88-1.15)		.95	.95
Baseline PROMIS, sleep		13,756	1.07 (0.97-1.19)	.19	.41	1.04 (0.91-1.20)		.55	.75	0.97 (0.86-1.11)		.70	.86
Baseline PROMIS, social		13,756	1.04 (0.95-1.13)	.41	.54	1.08 (0.92-1.26)		.38	.57	0.94 (0.83-1.07)		.36	.58
**Insurance plan type**		13,063	—	.94	.94	—		.19	.49	—		.76	.88
	Private	6621	Reference	—	—	Reference		—	—	Reference		—	—
	Mixed	1911	0.98 (0.63-1.53)	.94	—	0.55 (0.28-1.08)		.08	—	1.15 (0.51-2.62)		.73	—
	Public	4531	0.93 (0.64-1.36)	.73	—	0.70 (0.37-1.33)		.28	—	1.23 (0.70-2.17)		.47	—
**Cancer type (collapsed)**		13,954	—	.11	.34	—		.69	.85	—		.66	.86
	Gastrointestinal tract	4810	Reference	—	—	Reference		—	—	Reference		—	—
	Pancreas	3313	0.69 (0.46-1.04)	.08	—	0.75 (0.33-1.72)		.50	—	1.24 (0.65-2.36)		.51	—
	Breast	2158	1.25 (0.81-1.94)	.31	—	0.74 (0.36-1.52)		.41	—	1.54 (0.70-3.37)		.28	—
	Urogenital	1275	1.09 (0.59-2.03)	.78	—	0.48 (0.17-1.31)		.15	—	0.92 (0.37-2.32)		.87	—
	Other	2398	1.20 (0.74-1.95)	.45	—	0.76 (0.39-1.48)		.42	—	1.54 (0.75-3.15)		.24	—
**Cancer stage 4**		13,681	—	.25	.41	—		.91	.94	—		.52	.72
	No	4959	Reference	—	—	Reference		—	—	Reference		—	—
	Yes	8722	0.81 (0.56-1.16)	.25	—	1.03 (0.62-1.72)		.91	—	0.84 (0.51-1.41)		.52	—
Time since cancer diagnosis (months)		13,954	1.01 (1.00-1.01)	.18	.41	1.01 (0.99-1.02)		.23	.49	1.01 (1.00-1.02)		.21	.44
Study day		13,954	0.99 (0.99-0.99)	<.001	<.001	1.00 (0.99-1.00)		.27	.49	1.00 (0.99-1.00)		.03	.13
**Weekend**		13,954	—	<.001	.002	—		.20	.49	—		.007	.05
	No	9976	Reference	—	—	Reference		—	—	Reference		—	—
	Yes	3978	0.89 (0.84-0.95)	<.001	—	0.94 (0.86-1.03)		.20	—	0.93 (0.89-0.98)		.007	—
Time since last chemotherapy (days)		12,697	0.99 (0.98-0.99)	<.001	<.001	0.98 (0.97-1.00)		.02	.22	0.99 (0.98-0.99)		<.001	.002

^a^For smartphone adherence outcome, adjusted for phone type.

^b^OR: odds ratio.

^c^Unadjusted Wald test *P* value for single- or multi-parameter inference.

^d^Adjusted global Wald test *P* value, corrected for multiple comparisons.

^e^Not applicable.

^f^PROMIS: Patient-Reported Outcomes Measurement Information System.

^g^PROPr: PROMIS-Preference scoring system.

For the daily survey adherence outcome, there were statistically significant effects of race, weekends, time in the study, and time since last chemotherapy treatment. The odds of completing a daily survey were significantly lower for non-White or non-Caucasian participants relative to White or Caucasian participants (OR 0.48, 95% CI 0.29-0.80; *P*=.004), on weekend days relative to weekday days (OR 0.89, 95% CI 0.84-0.95; *P*<.001), with each additional day in the study following enrollment (OR 0.99, 95% CI 0.99-0.99; *P*<.001), and with each additional day since the participant’s last chemotherapy treatment (OR 0.99, 95% CI 0.98-0.99; *P*<.001).

For the smartphone adherence outcome, there were statistically significant effects of baseline depression and time since last chemotherapy treatment, after adjusting for phone type. Each 10 percentage-point increase (ie, an increase of 0.1) in baseline PROMIS preference depression subscale score, reflecting less depression, was associated with higher odds of adherence (OR 1.14, 95% CI 1.00-1.29; *P*=.046), while each additional day since the participant’s last chemotherapy treatment was associated with lower odds of adherence to smartphone data collection (OR 0.98, 95% CI 0.97-1.00; *P*=.02). These effects did not survive correction for multiple comparisons (baseline depression *Q*=.32, time since last chemotherapy treatment *Q*=.22).

For the Fitbit adherence outcome, there were statistically significant effects of age, race, weekends, time in the study, and time since last chemotherapy treatment. Odds of adherence to Fitbit data collection increased with each additional year of age relative to the mean age of the sample (OR 1.02, 95% CI 1.00-1.05; *P*=.03). Odds of adherence to Fitbit data collection were significantly lower for non-White or non-Caucasian participants relative to White or Caucasian participants (OR 0.36, 95% CI 0.19-0.68; *P*=.002), on weekend days relative to weekday days (OR 0.93, 95% CI 0.89-0.98; *P*=.007), with each additional day in the study following enrollment (OR 1.00, 95% CI 0.99-1.00; *P*=.03), and with each additional day since the participant’s last chemotherapy treatment (OR 0.99, 95% CI 0.98-0.99; *P*<.001). Effects of age (*Q*=.13), weekends (*Q*=.05), and time in the study (*Q*=.13) did not survive correction for multiple comparisons.

### Multivariable Models

To determine how a purposeful subset of these predictors were together associated with adherence, we fit a single multivariable GEE, separately for each data stream. Predictors chosen a priori included (1) age; (2) gender; (3) race; (4) education; (5) rural zip code; (6) baseline PROMIS preference scores, cognition and depression subscales; (7) stage 4 cancer; (8) study day; (9) weekends; and (10) time since last chemotherapy. As with the univariable models, we additionally adjusted for phone type in the model for the smartphone data collection adherence outcome only.

**Figure 1:**
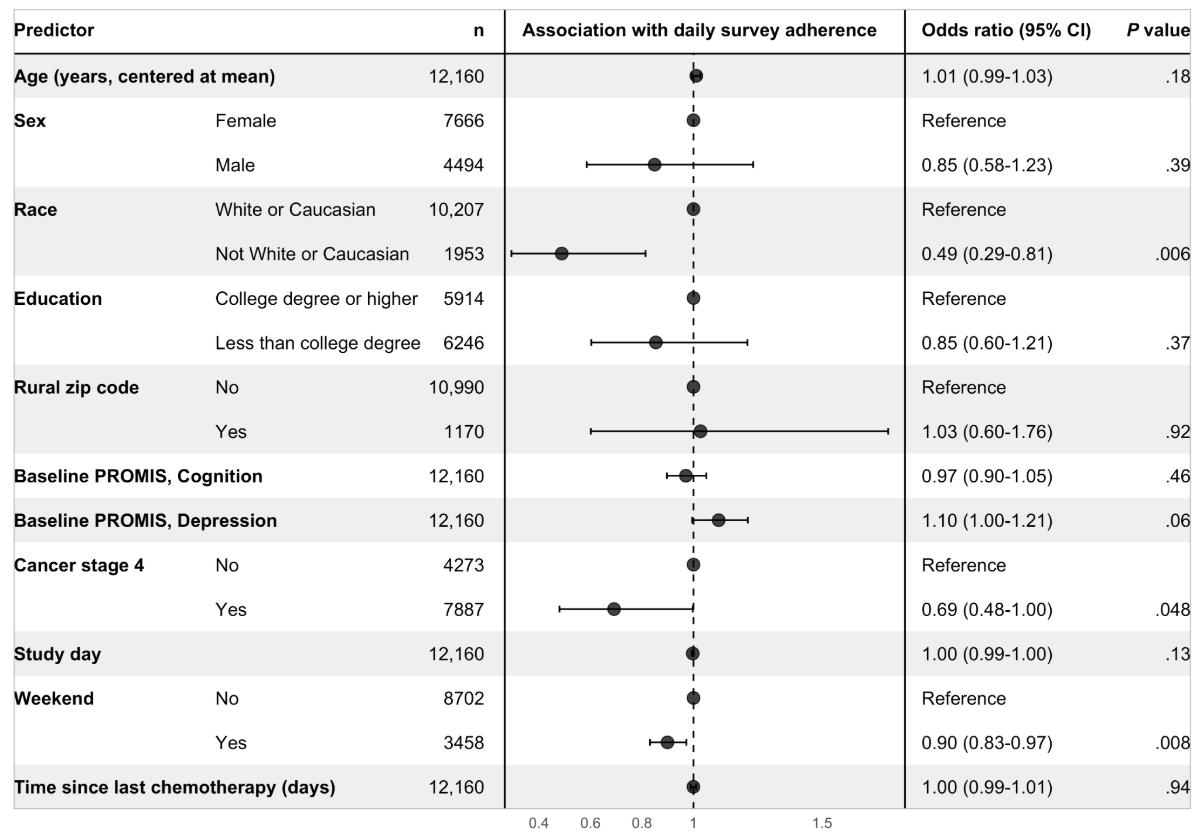
Results of the multivariable model for the daily survey adherence outcome. Each row corresponds to a predictor or predictor category, with separate predictors delineated by alternating gray and white bands. The center panel displays the adjusted odds ratio point estimate and 95% CI. Adjusting for other predictors in the model, odds of adherence to daily survey completion were significantly lower among non-White or Caucasian participants, participants with stage 4 cancer, and on weekend days. PROMIS: Patient-Reported Outcomes Measurement Information System.

Results of the multivariable models were generally consistent with those of the univariable models. For the daily survey adherence outcome (Figure 1), we again found that, adjusting for other predictors in the model, non-White or non-Caucasian participants were less likely to complete a daily survey relative to White or Caucasian participants (OR 0.49, 95% CI 0.29-0.81; *P*=.006), and participants were less likely to complete surveys on weekend days relative to weekday days (OR 0.90, 95% CI 0.83-0.97; *P*=.008). In addition, participants with stage 4 cancer were significantly less likely to be adherent to daily survey completion relative to participants with cancer in stages 0-3 (OR 0.69, 95% CI 0.48-1.00; *P*=.048). Controlling for the other predictors in the model, time in the study and time since last chemotherapy treatment were no longer significantly associated with daily survey adherence.

**Figure 2:**
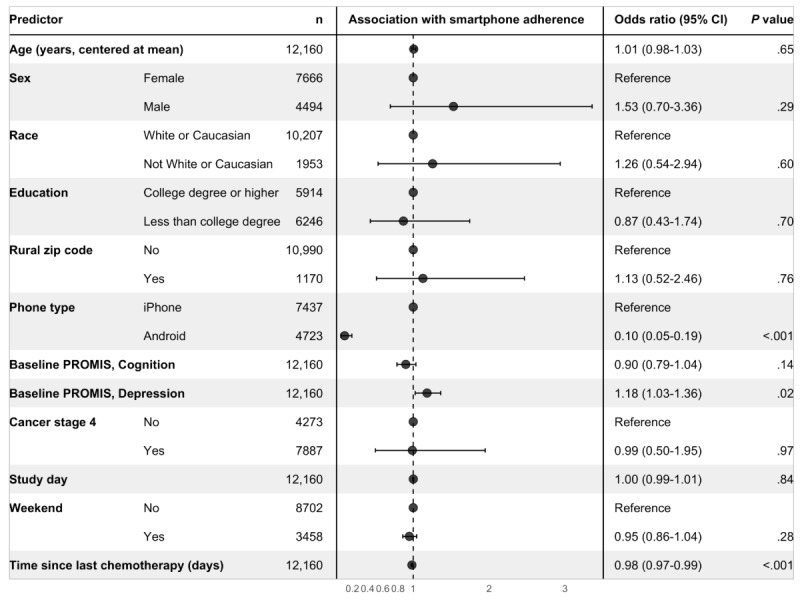
Results of the multivariable model for the smartphone adherence outcome. Each row corresponds to a predictor or predictor category, with separate predictors delineated by alternating gray and white bands. The center panel displays the adjusted odds ratio point estimate and 95% CI. Adjusting for other predictors in the model, odds of adherence to smartphone data collection were significantly lower among participants with Android devices and with each additional day since the participant’s last known chemotherapy treatment; odds of adherence were higher among participants with higher PROMIS depression subscale scores (reflecting less depression). PROMIS: Patient-Reported Outcomes Measurement Information System.

For the smartphone adherence outcome (Figure 2), we again found that there were significant effects of phone type, baseline depression, and time since last chemotherapy treatment. Relative to participants with iPhone devices, participants with Android devices were less likely to be adherent to smartphone data collection, defined as at least 8 valid hours of data collected from any AWARE sensor, due to differences in sampling rates across device platforms (OR 0.10, 95% CI 0.05-0.19; *P*<.001). In the adjusted model, each 10 percentage-point increase (ie, an increase of 0.1) in baseline PROMIS preference depression subscale score, reflecting less depression, was again associated with higher odds of adherence (OR 1.18, 95% CI 1.03-1.36; *P*=.02), while each additional day since the participant’s last chemotherapy treatment was associated with lower odds of adherence to smartphone data collection (OR 0.98, 95% CI 0.97-0.99; *P*=.001).

**Figure 3:**
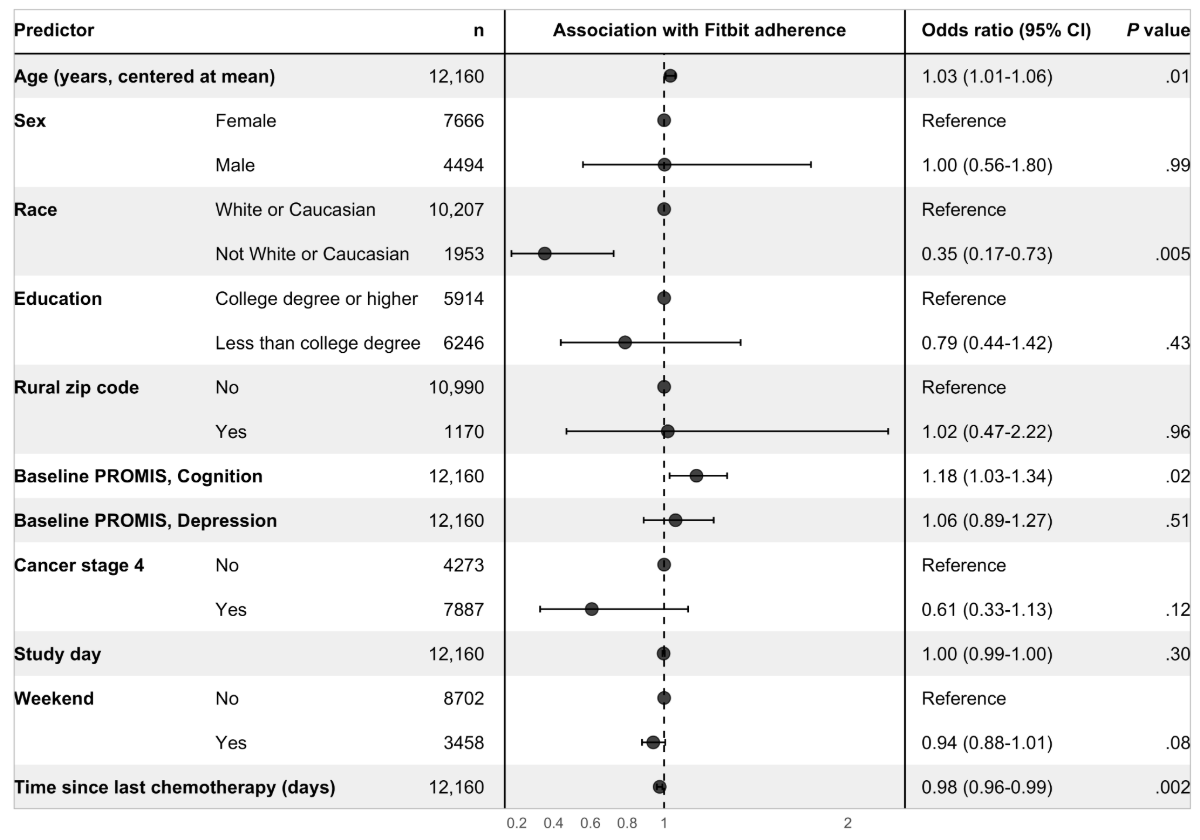
Results of the multivariable model for the Fitbit adherence outcome. Each row corresponds to a predictor or predictor category, with separate predictors delineated by alternating gray and white bands. The center panel displays the adjusted odds ratio point estimate and 95% CI. Adjusting for other predictors in the model, odds of adherence to Fitbit data collection were significantly lower among non-White or Caucasian participants and with each additional day since the participant’s last known chemotherapy treatment; odds of adherence were higher among older participants and those with higher PROMIS cognitive subscale scores (reflecting better cognitive abilities). PROMIS: Patient-Reported Outcomes Measurement Information System.

For the Fitbit adherence outcome (Figure 3), we again found that there were significant effects of age, race, and time since last chemotherapy treatment. Odds of adherence to Fitbit data collection increased with each additional year of age relative to the mean age of the sample (OR 1.03, 95% CI 1.01-1.06; *P*=.01). Odds of adherence to Fitbit data collection were significantly lower for non-White or non-Caucasian participants relative to White or Caucasian participants (OR 0.35, 95% CI 0.17-0.73; *P*=.005) and with each additional day since the participant’s last chemotherapy treatment (OR 0.98, 95% CI 0.96-0.99; *P*=.002). In addition, adjusting for other predictors in the model, there was a significant effect of baseline cognition, with each 10 percentage-point increase in baseline PROMIS preference cognition subscale score (ie, an increase of 0.1), reflecting better cognitive abilities, associated with 18% higher odds of adherence to Fitbit data collection (OR 1.18, 95% CI 1.03-1.34; *P*=.02). Controlling for other predictors, the effects of time in the study and weekends were no longer statistically significant.

## Discussion

### Principal Findings

To our knowledge, this is the first study to examine participant engagement with multiple, concurrent methods of remote patient monitoring during chemotherapy, including daily symptom reporting through smartphone, passive smartphone sensing, and wearable device data collection over a 90-day observational study. In addition, our study examined the effects that different sociodemographic, quality of life, clinical, and time-related factors had on patient engagement with each of the 3 different data streams.

Overall adherence rates support the feasibility of mobile technology–based data collection during chemotherapy, with higher rates of adherence to both smartphone sensing and wearable data collection relative to daily symptom surveys. This is likely because the daily symptom surveys required active engagement from the participant compared with the passive smartphone and wearable data collection. Upon enrollment, a member of our team worked with each participant individually to set up their Fitbit and ensure that it was working properly, which may have contributed to this difference. However, adherence metrics were lower for some participants due to various technical issues (eg, AWARE app crashing, phone being broken or replaced, and Fitbit device not syncing automatically). Overall, our results are consistent with other studies that have shown that daily symptom reporting and continuous collection of wearable device data are feasible during chemotherapy [8-15]. In our study, overall engagement with daily symptom reporting (61%) fell between adherence rates observed among other symptom tracking studies (55%-83%) [9,17], and overall adherence to collecting wearable device data (70%) similarly fell between those previously observed (45%-85%) [13,15].

Our results suggest that adherence varied based on demographic factors (age and race), clinical factors (cancer stage and patient-reported depression and cognition), and timing (including days since last chemotherapy treatment, time in study, and weekend vs weekday). Relative to White participants, non-White patients demonstrated lower levels of engagement with both the daily symptom surveys and wearable data collection, suggesting that we need new methods of engaging patients with cancer from racial and ethnic minority groups in technology-based monitoring. Patients with stage 4 cancer exhibited lower rates of engagement with daily symptom reporting than those with earlier stage disease, likely due to greater disease burden and associated life disruption. In addition, time since last chemotherapy treatment was associated with both smartphone and wearable device engagement, with participants more likely to engage with both data streams the less time that had elapsed since their last chemotherapy treatment. This association was likely due to several factors, including coordinators being able to meet with participants and troubleshoot technology difficulties during treatments. The treatments also likely served as reminders for participants to engage with the study. Consistent with previous work, participants were also less likely to complete symptom surveys on weekend days relative to weekdays [17]. Patients may have more “routine” schedules during the week, and thus are more easily able to remember to fill out the surveys. In addition, many participants had work responsibilities during the week, and thus interference related to symptoms such as fatigue may have been more salient and served as a reminder to report their symptoms.

One surprising finding was that older age was associated with better adherence to wearable device data collection, which contradicts beliefs that older adults are less likely to adopt or engage with health technology. There remains a false belief within the scientific community that older patients are unable or unwilling to engage with digital health assessments and interventions [35]. Unfortunately, due to this stigma, there is a relative lack of research in this patient population regarding their engagement with mobile health technology. Perhaps older participants, who may have been less likely than younger participants to be working or caring for children, had fewer competing demands on their time and attention and were better able to focus on the research project. We also found that better self-reported cognitive abilities at study entry predicted greater engagement with wearable Fitbit data collection, suggesting that while older adults may be more adherent, additional reminders or strategies may need to be implemented to support patients with any cognitive impairments in collecting wearable data. Interestingly, a study among patients with breast and prostate cancer of engagement levels with a symptom tracking app, similar to the survey app used in our study, also showed a positive association between age and engagement [9].

### Limitations

This study is not without limitations. First, participants needed to own a smartphone that was compatible with study apps and be able to read and write in English to enroll in the study. This likely skewed our sample population to be more “tech literate” than the general population of patients receiving chemotherapy. Second, there was likely a selection bias present in our sample, as participants who were less likely to engage in our study would be more likely to decline enrollment. Third, we assessed engagement in the context of a research study where we were monitoring incoming data closely and reaching out to participants to troubleshoot technical or compliance issues frequently; it is likely that we would have lower rates of engagement without these interactions with research staff. Fourth, a participant’s day-to-day symptom burden may have affected their survey response rate. Participants may have been more or less likely to fill out the surveys on days where they had particularly high (or low) symptom burden, which could skew our results; future research should examine the association between symptom burden and patient-reported outcome completion. We set a priori thresholds based on the literature and our previous work to define a day as having a completed survey (at least 50%) or sufficient wearable or smartphone sensor data (at least 8 hours), and studies that select different thresholds may draw different conclusions. We were lacking information about cancer treatments, including information about chemotherapy type and dose as well as additional treatments patients may have been receiving, such as immunotherapy or targeted therapy. The different findings observed for participants with iOS versus Android smartphones suggest there may be significant measurement bias in smartphone sensing and differences in how each operating system collected smartphone sensor data. Finally, it is important to note that the remote assessments collected as part of the current study were not shared with clinicians or used to inform clinical care, and participants were advised upon consent that data would not be shared or accessed by their clinicians. This is different from other symptom monitoring studies that incorporated clinician alerts or other communication with the care team [7]. Participants may be more motivated to engage with remote technology-based assessments when they know this information is being used to guide their cancer care. Future studies should also explore the feasibility of similar data collection methods in broader populations, including adolescents and young adults with cancer, patients receiving other forms of cancer treatment (radiation, immunotherapy, etc), and patients with nonsolid tumor cancers.

### Conclusion

Despite these limitations, our study showed feasible levels of engagement with all 3 of our data streams over 90 days. These results demonstrate that collecting patient-reported symptom ratings through smartphone, passive smartphone sensor data, and wearable device data over long periods of time is feasible in cancer trials, even among older patients and patients with advanced cancer receiving active treatment. Findings provide some support for the idea that the digital divide may widen existing health disparities, with non-White participants demonstrating lower levels of engagement, but also challenge the idea that older adults will be less likely to adopt or engage with technology, as least with regard to wearable devices. Future work should experiment with different ways of optimizing engagement for all groups, including different delivery formats and schedules of reminders, onboarding and training procedures, and levels of integration with the clinical care team. More pragmatic studies should also explore levels of engagement with symptom reporting and other patient-generated health data collection in the context of routine clinical care, without research staff monitoring or intervening with participants. To our knowledge, this is the first study to examine patterns and predictors of participant engagement with daily symptom reporting, smartphone sensing, and wearable device data collection during outpatient chemotherapy, and results provide encouragement and guidance for additional work in this area.

## Abbreviations

ar1: first-order autoregressive
EMR: electronic medical record
GEE: generalized estimating equations
OR: odds ratio
PROMIS: Patient-Reported Outcomes Measurement Information System
PROPr: PROMIS-Preference scoring system
QIC: quasi-information criterion
RAPIDS: Reproducible Analysis Pipeline for Data Streams

## Data Availability

The datasets generated and analyzed during this study are available from the corresponding author on reasonable request.
